# Reconstruction and Evaluation of the Synthetic Bacterial MEP Pathway in *Saccharomyces cerevisiae*

**DOI:** 10.1371/journal.pone.0052498

**Published:** 2012-12-28

**Authors:** Siavash Partow, Verena Siewers, Laurent Daviet, Michel Schalk, Jens Nielsen

**Affiliations:** 1 Department of Chemical and Biological Engineering, Chalmers University of Technology, Göteborg, Sweden; 2 Firmenich SA, Corporate R&D Division, Geneva, Switzerland; Texas A&M, United States of America

## Abstract

Isoprenoids, which are a large group of natural and chemical compounds with a variety of applications as e.g. fragrances, pharmaceuticals and potential biofuels, are produced via two different metabolic pathways, the mevalonate (MVA) pathway and the 2-C-methyl-D-erythritol 4-phosphate (MEP) pathway. Here, we attempted to replace the endogenous MVA pathway in *Saccharomyces cerevisiae* by a synthetic bacterial MEP pathway integrated into the genome to benefit from its superior properties in terms of energy consumption and productivity at defined growth conditions. It was shown that the growth of a MVA pathway deficient *S. cerevisiae* strain could not be restored by the heterologous MEP pathway even when accompanied by the co-expression of genes *erpA*, *hISCA1* and *CpIscA* involved in the Fe-S trafficking routes leading to maturation of IspG and IspH and *E. coli* genes *fldA* and *fpr* encoding flavodoxin and flavodoxin reductase believed to be responsible for electron transfer to IspG and IspH.

## Introduction

In connection with the production of many natural products the transfer of complete biosynthetic pathways from native to heterologous organisms is an attractive approach, as it may allow for use of industrially compatible strains and for further pathway engineering [1], [2], [3]. Although this approach imposes a number of challenges such as gene codon optimization, correct protein folding and proper enzyme function, there are a several examples where whole biochemical pathways have been transferred successfully such as expressing the mevalonate (MVA) pathway in *Escherichia coli* [4], transferring a complex mammalian hydrocortisone biosynthetic pathway containing 8 genes into yeast [5], and re-construction of the early four steps of the flavonoid biosynthetic pathway in *Saccharomyces cerevisiae* in order to convert phenylpropanoid acids into flavanones [6].

The MVA pathway in yeast and most other eukaryotes and the 2-C-methyl-D-erythritol (MEP) pathway in most bacteria and plant plastids are responsible for production of isoprenoids, which represent an important class of biochemical compounds [7]. The MEP pathway was first reported independently by Rohmer and Argoni [8], [9]. This pathway initiates by condensation of one molecule each of pyruvate and D-glyceralaldehyde-3-phosphate through a thiamin diphosphate dependent reaction catalyzed by 1-deoxy-D-xylulose 5-phosphate synthase (Dxs) [10], followed by an NADPH dependent reduction process being catalyzed by 1-deoxy-D-xylulose 5-phosphate reductoisomerase (Dxr) [11], generating 2-C-methyl-D-erythritol 4-phosphate (MEP). This intermediate is converted into the cyclic 2,4-diphosphate of 2-C-methyl-D-erythritol by the sequential action of the enzymes specified by IspD, IspE and IspF [12], [13], [14]. 2-C-methyl-D-erythritol-2,4-cyclodiphosphate is reduced by a reductase encoded by the *ispG* gene [15], [16] followed by the production of IPP and DMAPP by the action of the *ispH* gene product [17], [18]. Unlike the MVA pathway, the MEP pathway has not been investigated extensively, in particular in heterologous hosts. *S. cerevisiae* is widely used as a platform for heterologous expression of biochemical pathways [5], [19], [20], due to its well-characterized physiology and the availability of molecular biology tools. Maury and co-workers reported the reconstruction of the bacterial MEP pathway in *S. cerevisiae* by expression of seven enzymatic steps of the pathway from self-replicating, high-copy yeast plasmids [21]. By inhibiting the endogenous MVA pathway through addition of lovastatin, it was shown that the MEP pathway was active and could ensure production of ergosterol, which is essential for yeast. However, transferring entire biochemical pathways using episomal plasmids is not recommended for industrial applications due to poor genetic stability. In addition, maintenance of plasmids requires selective pressure provided by selective media which increase the costs. In contrast, gene integration offers a stable manipulation without requirement of selective pressure provided through the media.

In this work, we show by using genome-scale modeling that transferring the entire bacterial MEP pathway into *S. cerevisiae* gives a higher theoretical maximum yield of the isoprenoid precursor compared with biosynthesis via the endogenous MVA pathway. In order to activate this pathway in yeast eight enzymatic steps of the bacterial MEP pathway were integrated into the chromosome of *S. cerevisiae*. Following expression of the heterologous MEP pathway, we found that the IspG and the IspH enzymes are potential bottlenecks of the MEP pathway in *S. cerevisiae* and activating them requires the successful transfer of Fe-S clusters to these two enzymes and a suitable electron transfer system. Therefore, both possible Fe-S trafficking routes responsible for maturation of IspG and IspH and a bacterial electron transfer system were re-constructed in the yeast cytosol by co-expression of the bacterial gene *erpA* with *iscA* from either human or *Arabidopsis thaliana* and flavodoxin and flavodoxin reductase, respectively. These genetic modifications were accompanied with over-expression of *IspG* and *IspH* from *A. thaliana.* However, introducing the above mentioned manipulations did not result in a functional MEP pathway in *S. cerevisiae*.

Based on this study we suggest that specific physical interaction or compartmentalization is required for *in vivo* biogenesis and transfer of essential prosthetic groups, in this case transfer of iron-sulfur clusters, into apoIspG and apoIspH and consequently activation of the bacterial MEP pathway.

## Results

### *In silico* Evaluation of MVA and MEP Pathway in *S. cerevisiae*

**Figure 1:**
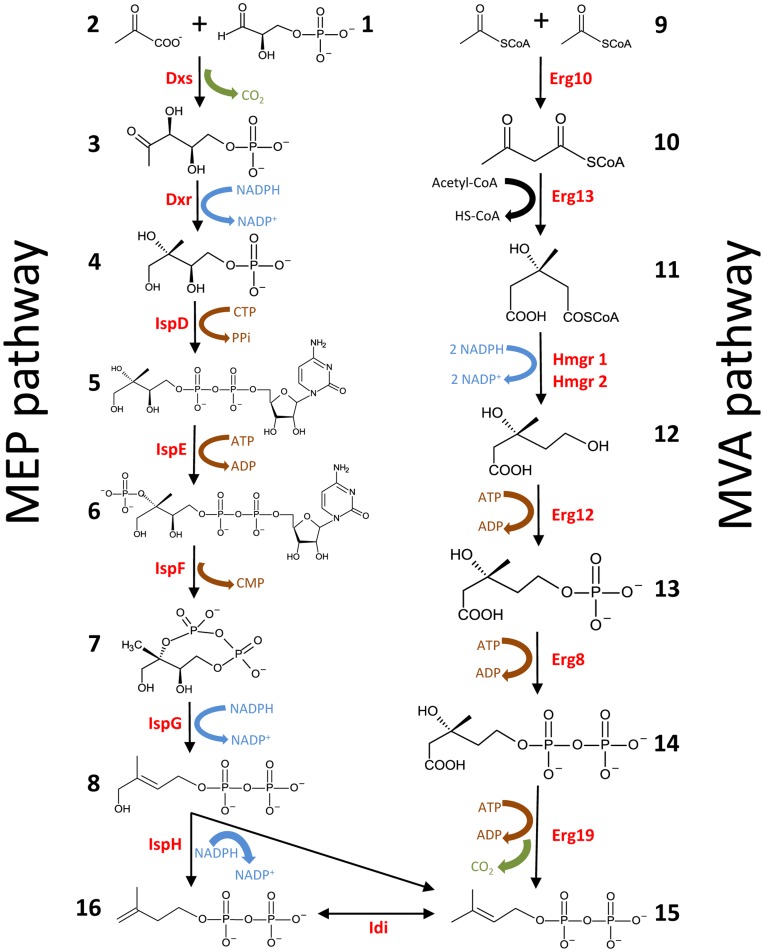
The MEP pathway (left). Enzymes: **Dxs,** 1-deoxy-D-xylulose-5-phosphate synthase; **Dxr,** 1-deoxy-D-xylulose 5-phosphate reductoisomerase; **IspD,** 4-diphosphocytidyl-2-C-methyl-D-erythritol synthase; **IspE,** 4-diphosphocytidyl-2-C-methylerythritol kinase; **IspF,** 2-C-methyl-D-erythritol 2,4-cyclodiphosphate synthase; **IspG,** 4-hydroxy-3-methylbut-2-en-1-yl diphosphate synthase; **IspH,** 1-hydroxy-2-methyl-butenyl 4-diphosphate reductase; Metabolites: **1,** D-glyceraldehyde 3-phosphate; **2,** pyruvate; **3,** 1-deoxy-D-xylulose 5-phosphate; **4,** 2-C-methyl-D-erythritol 4-phosphate; **5,** 4-diphosphocytidyl-2-C-methyl-D-erythritol; **6,** 2-phospho-4-diphosphocytidyl-2-C-methyl-D-erythritol; **7,** 2-C-methyl-D-erythritol 2,4-cyclodiphosphate; **8,** 1-hydroxy-2-methyl-2-(E)-butenyl 4-diphosphate. The MVA pathway (right). Enzymes: **Erg10,** acetoacetyl-CoA thiolase; **Erg13,** 3-hydroxy-3-methylglutaryl-CoA synthase; **Hmg1/2,** 3-hydroxy-3-methylglutaryl-CoA reductase; **Erg12,** mevalonate kinase; **Erg8,** phosphomevalonate kinase; **Erg19,** mevalonate diphosphate decarboxylase; **Idi,** isopentenyl diphosphate isomerase; Metabolites: **9,** acetyl-CoA; **10,** acetoacetyl-CoA; **11,** 3-hydroxy-3-methylglutaryl-CoA; **12,** mevalonate; **13,** phosphomevalonate; **14,** diphosphomevalonate; **15,** isopentenyl diphosphate; **16,** dimethyl allyl diphosphate.

Seven genes responsible for the enzymatic steps of the bacterial MEP pathway (Figure 1) were introduced into the yeast genome scale metabolic model iIN800 [22]. The efficiency of the MEP pathway was evaluated and compared with the endogenous MVA pathway using the model. The model was optimized for maximum production of farnesyl pyrophosphate (FPP), which is a branch point intermediate in ergosterol biosynthesis, for two different conditions, using the endogenous MVA pathway and using the heterologous MEP pathway, respectively. The result showed that by consuming 1 mol of glucose 0.21 and 0.24 mol farnesyl pyrophosphate could be produced through the MVA and MEP pathway, respectively. According to this analysis the FPP production through the MEP pathway results in a favorable theoretical yield.

The stoichiometry calculation of NAD(P)H and ATP consumption for both pathways showed that for producing one molecule of farnesyl pyrophosphate from glucose via the MVA pathway six molecules of NADPH and nine molecules of ATP are required, while production via the MEP pathway consumes nine molecules of NAD(P)H and six molecules of ATP. Provision of sufficient cytosolic NADPH is therefore a critical factor for both pathways. In contrast to the MEP pathway, which consumes only 3 molecules of glucose, the MVA pathway consumes 4.5 molecules of glucose for the biosynthesis of one molecule farnesyl pyrophosphate (these values are excluding use of glucose for production of ATP and redox co-factors).

Combining the results derived from the yeast genome scale metabolic model and the stoichiometry calculation, it became evident that the MEP pathway is a more efficient route than the endogenous MVA pathway for isoprenoid production in terms of energy consumption and productivity. Therefore we decided to investigate this pathway in *S. cerevisiae*, which has been widely used as a platform for heterologous expression of isoprenoids [19], [20].

### Genomic Integration of MEP Pathway Genes

**Figure 2:**
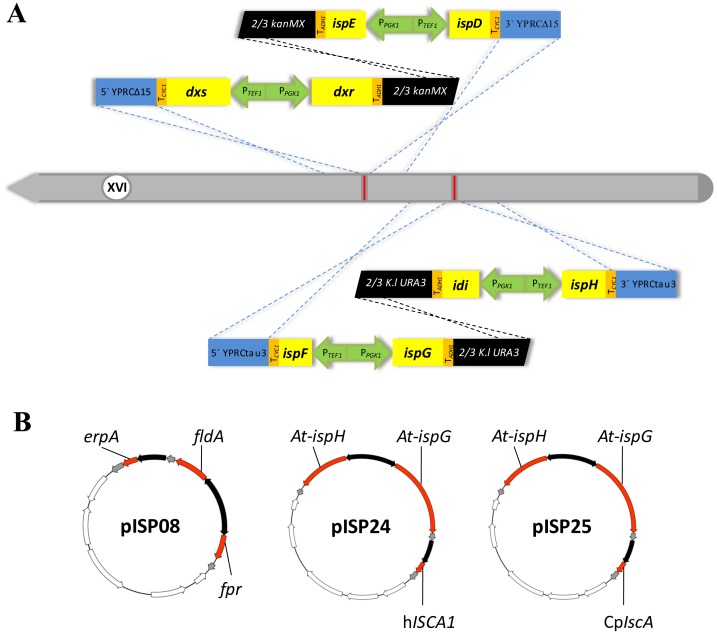
Schematic representation of genetic engineering strategies for A) genomic integration of the bacterial MEP pathway genes into the yeast genome (chromosome XVI), and B) plasmid-based reconstruction of possible Fe/S trafficking routes involved in maturation of bacterial IspG/IspH, bacterial electron transfer systems and plant-derived *ispG/ispH* in *S. cerevisiae.* For details see text.

Since the efficiency of *in vivo* homologous recombination in *S. cerevisiae* is high, a bipartite integration strategy was applied [23]. Eight codon optimized MEP pathway genes including *dxs, dxr, ispD, ispE, ispF, ispG, ispH* and *idi* were organized in four different synthetic fragments (Figure 2A). Each fragment contained two genes located on each side of the bidirectional promoter P*_TEF1_-*P*_PGK1_*, which had shown high constitutive activity in glucose containing media before [24], and in front of either the *ADH1* or the *CYC1* terminator. Furthermore, each fragment harbored a part (ca. 2/3) of a gene coding for a selectable marker (*kanMX* or *K.l.URA3*) and flanking regions which corresponded to the desired integration site on the chromosome and which were necessary for integration via homologous recombination. In order to recycle the selectable markers, direct repeat DNA sequences of 143 bp were introduced at both sides of *K.l.URA3*, whereas *loxP* sites flanked the *kanMX* cassette.

Previously, different transcription levels among various chromosomal regions in *S. cerevisiae* have been reported by using *lacZ* as a reporter gene [25]. We have shown that the two integration sites, YPRCΔ15 and YPRCτ3, on chromosome XVI of *S. cerevisiae* provided potentially higher expression levels than other regions tested [25]. Therefore, all genes involved in the bacterial MEP pathway were integrated into these two sites in two steps (Figure 2A). The selectable markers, *kanMX* and *K.l*.*URA3*, were looped out. Integration and transcription of the MEP genes was confirmed by PCR and RT-PCR, respectively (data not shown). Table 1 lists the strains which were constructed during this work. Strains SCISP06 and SCISP12 were obtained through the integration of MEP pathway genes into the chromosome of CEN.PK 113-13D and CEN.PK 113-1C, respectively.

**Table 1:** List of strains and plasmids used in this study.

Strain	Genotype	Plasmid	Reference
**CEN.PK 113-13D**	*MATα MAL2-8c SUC2 ura3-52*	None	P. Kötter^1^
**SCISP06**	*MATα MAL2-8c SUC2 ura3-52 dxs dxr ispD ispE ispF* *ispG ispH idi*	None	this work
**SCISP16**	*MATα MAL2-8c SUC erg13::loxP-KanMX-loxP dxs dxr ispD* *ispE ispF ispG ispH idi*	pISP08	this work
**SCISP28**	*MATα MAL2-8c SUC2 erg13::loxP-KanMX-loxP,*	pSP-GM1	this work
**SCISP29**	*MATα MAL2-8c SUC2 erg13::loxP-KanMX-loxP dxs dxr* *ispD ispE ispF ispG ispH idi*	pSP-GM1	this work
**CEN.PK 113-1C**	*MATa MAL2-8c SUC2 trp1-289 ura3-52 his3Δ1*	None	P. Kötter^1^
**SCISP12**	*MATa MAL2-8c SUC2 trp1-289 ura3-52 his3Δ1 dxs dxr* *ispD ispE ispF ispG ispH idi*	None	this work
**SCISP13**	*MATa MAL2-8c SUC2 trp1-289 his3Δ1 dxs dxr ispD ispE ispF ispG ispH idi*	pISP08^2^	this work
**SCISP30**	*MATa MAL2-8c SUC2 trp1-289 erg13::loxP-KanMX-loxP*	pSP-GM1, pSP-GM3	this work
**SCISP31**	*MATa MAL2-8c SUC2 trp1-289 erg13::loxP-KanMX-loxP* *dxs dxr ispD ispE ispF ispG ispH idi*	pISP08, pISP24^3^	this work
**SCISP32**	*MATa MAL2-8c SUC2 trp1-289 erg13::loxP-KanMX-loxPdxs* *dxr ispD ispE ispF ispG ispH idi*	pISP08, pISP25^4^	this work

1 University of Frankfurt, Germany.

2 pISP08 contains *erpA, fpr* and *fldA.*

3 pISP24 contains *hISCA1, At-IspG* and *At-IspH*.

4 pISP25 contains *CpIscA, At-IspG* and *At-IspH*.

**Figure 3:**
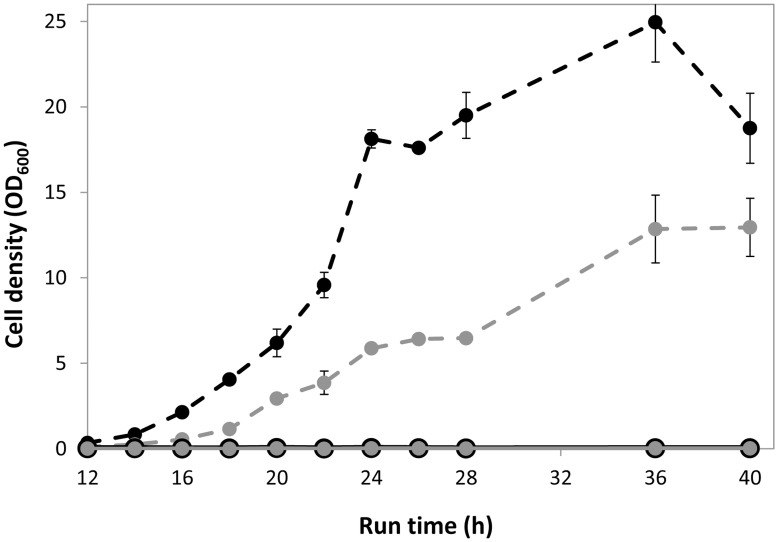
Growth of *S. cerevisiae* strains CEN.PK 113-13D (black circles) and SCISP06 (gray circles) in SD minimal medium. Dashed lines represent the growth in 0 g L^−1^ of lovastatin; solid lines represent the growth in presence of 2 g L^−1^ of lovastatin. Error bars show the standard deviation from three cultivations.

The functionality of the bacterial MEP pathway was tested by blocking the endogenous MVA pathway which is essential for *S. cerevisiae* because of its supply of ergosterol involved in regulation of membrane fluidity [26]. The MVA pathway was inhibited using lovastatin (mevinolin) [27] which is a therapeutic agent and acts as a competitive inhibitor of an early pathway enzyme, HMG-CoA reductase. Surprisingly, no growth was observed in both wild type (CEN.PK 113-13D) and SCISP06 (yeast strain with integrated MEP pathway) in presence of 2 g L^−1^ lovastatin (Figure 3), respectively, which showed that the MEP pathway could not complement the MVA pathway, and which is in contrast to the previous report [21].

### Re-construction the Possible Bacterial Fe/S Trafficking Routes and the Bacterial Electron Transfer System

The detection of intermediates 3 and 5 (Figure 1) in the MEP-pathway carrying yeast strains indicated proper activity of the Dxs, Dxr and the IspD enzymes (data not shown). In addition, no-activity was observed for the last two enzymes of the MEP pathway, IspG and IspH, when expressed in yeast in a previous study [28]. IspG and IspH are known to be iron-sulfur cluster proteins [15], [16], [17], [29], [30], [31] and it has been reported that this cluster is directly involved in IspH activity [32]. The essential role of ErpA, which is an A-type iron-sulfur cluster protein, in the maturation process of IspG, and probably IspH, in *E. coli* has been investigated [33]. Furthermore, Puan and co-workers [34] identified *fldA* as an essential gene for isoprenoid biosynthesis in *E. coli*, as it provides reducing equivalents for the Fe/S clusters of IspG and IspH. *FldA* encodes flavodoxin I, which together with *fpr* encoded flavodoxin reductase composes an *E. coli* electron transfer system [35]. As an attempt to solve the problem of the non-functionality of the MEP pathway in *S. cerevisiae*, the impact of the co-expression of genes involved in transferring Fe-S clusters to IspG/IspH apoproteins and of the described *E. coli* electron transfer system was investigated. The coding region of genes *erpA, fpr* and *fldA* from *E. coli* were cloned on a single plasmid, pISP08, (Figure 2B) that was transformed into SCISP06 generating SCISP16 (Table1). The empty plasmid pSP-GM1 was transformed into CEN.PK113-13D and SCISP06 resulting in SCISP28 and SCISP29, respectively (Table 1).

**Figure 4:**
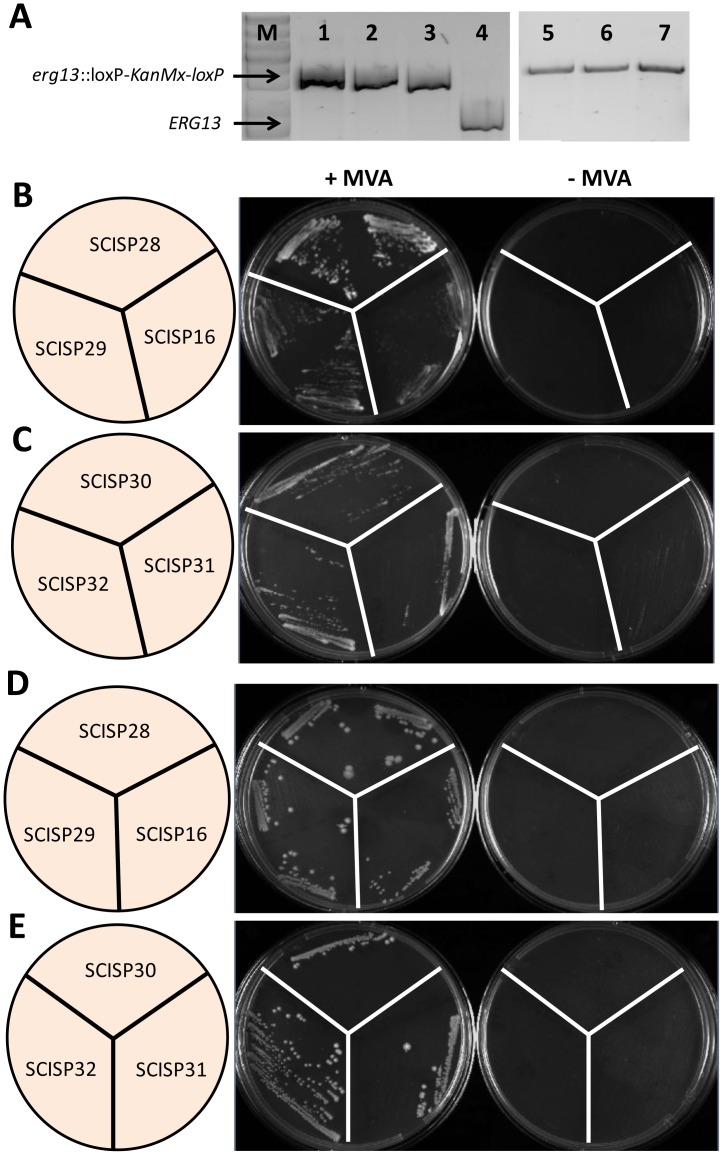
Schematic representation of evaluation the functionality of the bacterial MEP pathway in *S. cerevisiae* in different conditions. **A)** Gel electrophoresis of PCR products to confirm deletion of *ERG13* (1: SCISP28, 2: SCISP29, 3: SCISP16, 4: CEN.PK 113-13D (wild type), 5: SCISP30, 6: SCISP31, 7: SCISP32, M: 1 kb Plus DNA ladder (Fermentas, Maryland, USA); **B)** Aerobic cultivation of MEP pathway strains co-expressing *erpA, fpr and fldA*; **C)** Aerobic cultivation of MEP pathway strains co-expressing *erpA, fpr, fldA*, *At*-*IspG*, *At*-*IspH* with either *CpIscA* or *hISCA1*; **D)** Anaerobic cultivation of MEP pathway strains co-expressing *erpA, fpr and fldA*; E) Anaerobic cultivation of MEP pathway strains co-expressing *erpA, fpr, fldA*, *At*-*IspG*, *At*-*IspH* with either *CpIscA* or *hISCA1.* All strains carried an *ERG13* deletion and were plated on medium with or without 10 mg L^−1^ mevalonate (MVA).

To rule out any possible additional effect on cell growth using lovastatin for inhibition of the MVA pathway the functionality of the bacterial MEP pathway was investigated by deletion of *ERG13*, an essential gene in the MVA pathway. *ERG13* encodes HMG-CoA synthase [7], and its disruption results in a strain that requires exogenous mevalonate supplementation for viability [36]. The coding region of *ERG13* in strains SCISP16, SCISP28 and SCISP29 was replaced by a *kanMX* integration cassette, which was confirmed by PCR (Figure 4A). As it is illustrated in Figure 4B, the *ERG13* deleted strains could not grow in media lacking mevalonate under aerobic conditions.

**Figure 5:**
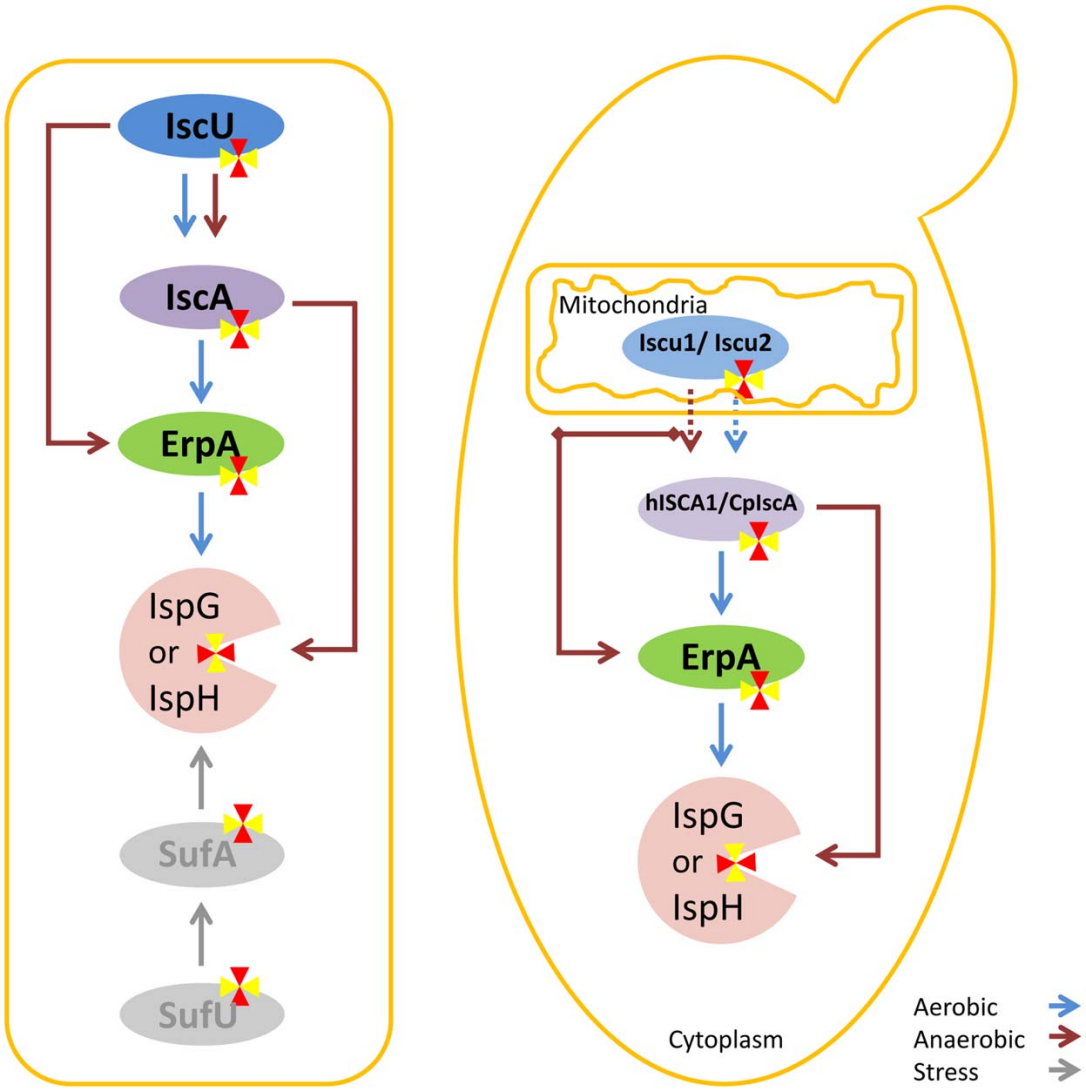
Schematic representation of possible Fe/S trafficking routes involved in maturation of bacterial IspG/IspH in *E. coli* (left) and reconstruction of possible routes preformed in this study in the yeast cytosol (right). Dashed arrows represent unknown mechanisms for transferring the Fe-S clusters from mitochondria to cytosol. For more information see text.

Since bacterial IspG and IspH have not shown any activity in yeast [28], we asked whether a eukaryotic version of both IspG and IspH would be active in yeast; the codon optimized plant genes of IspG and IspH from *A. thaliana* were chemically synthesized. In addition, an Fe-S trafficking model has previously been proposed that describes the transfer of Fe-S clusters to IspG and IspH in *E. coli* (Figure 5) [37]. The authors suggested that depending on the environmental conditions e.g. aerobic, anaerobic or stress, the Fe-S cluster is transferred from IscU or SufU scaffolds to apoIspG and apoIspH through the combination of A-type carriers including ErpA, IscA and SufA [37]. Assuming that this model can be transferred from *E. coli* to yeast the expression of *iscA* may fill the gap in this proposed model [37]. Therefore, one copy of each *IspG* and *IspH* from *A. thaliana* was cloned into expression plasmids with *iscA* from either human or *A. thaliana* resulting in pISP24 and pISP25, respectively (Figure 2B). Previously, localization and activity of human ISCA1 (hISCA1) was shown in mitochondria as well as in the cytosol of HeLa cells [38]. The authors have also demonstrated interaction of the small domain of IOP1 (**I**ron-**o**nly hydrogenase-like **p**rotein **I**) with human ISCA1 using yeast two-hybrid systems [38]. CpIscA from *A. thaliana* is involved in Fe-S biogenesis in chloroplasts [39]. The Fe-S cluster in CpIspA indicated stability in presence of oxygen [39]. Strains SCISP31 and SCISP32 were constructed by co-transforming pISP08 with either pISP24 or pISP25 into SCISP12, respectively (Table 1). Like for SCISP16, no growth was observed in the absence of exogenous mevalonate when *ERG13* was disrupted in both SCISP31 and SCISP32 (Figure 4C).

Fe-S clusters are sensitive to superoxide (O_2_^−^) and other oxidative agents [40], [41]. In addition, the Fe-S cluster of IspH is easily destroyed by exposure to molecular oxygen or other oxidative agents [32]. Therefore, to prevent inactivation of the Fe-S clusters in IspG and IspH, all *erg13* strains were also evaluated under anaerobic conditions. Yeast growing in anaerobic condition is ergosterol-dependent as the biosynthetis of ergosterol is disrupted in this condition. Therefore, exogenous ergosterol was added to the SD media at a final concentration of 1 mg L^−1^. None of the *erg13* strains showed mevalonate-independent growth (Figure 4D and 4E). This means that even in anaerobic conditions, the MEP pathway was not able to complement the MVA pathway.

## Discussion

In the current study, the seven enzymatic reactions of the bacterial MEP pathway were evaluated *in silico,* using the yeast genome scale metabolic model, iIN800 [22]. iIN800 consists of 1446 metabolic reactions and 1013 metabolites [22]. In comparison to the first *S. cerevisiae* model by Förster and co-workers [42], iIN800 covers lipid metabolism in great detail. Applying mathematical models has previously been demonstrated to be predictive and beneficial for metabolic engineering approaches e.g. to identify new target genes enhancing the biosynthesis of sesquiterpenes in yeast [43], and to schematically represent the effect of the interaction of protease supplementation and type of sugar on amino acid metabolisms in brewer’s yeast [44]. Here, *in silico* analysis of the bacterial MEP pathway expressed in yeast revealed the benefits of this heterologous pathway in terms of energy consumption and yield compared to the endogenous MVA pathway. Our result is in consistence with previous reports about the higher efficiency of the MEP pathway in converting sugars or glycerol into terpenoids compared to the MVA pathway [45], [46]. This is the rationale for our attempt to express the bacterial MEP pathway in yeast for production of isoprenoids.

The first step of most yeast metabolic engineering and synthetic biology studies involves re-construction of a complete or partially synthetic pathway. Although several methods have been developed [3], [47], *in vitro* DNA synthesis offers a fast, cheap and efficient method for synthesis of large DNA sequences [48], [49]. Besides, using synthetic genes with the possibility to manipulate codon bias can provide better control of the expression of heterologous MEP pathway genes in yeast. From the genetic engineering point of view, the codon bias is one of the first barriers in heterologous protein expression [50] and it can prevent the efficient biosynthesis of a recombinant protein because of altering the correlation between the frequency of the codon and the abundance of its corresponding tRNA, which impairs the translation machinery of the host [51]. The high efficiency and ease to work with *in vivo* homologous recombination in *S. cerevisiae* allows stable manipulation without requiring selective pressure for maintenance. Here we developed a strategy for easy integration of eight heterologous genes. The four DNA constructs containing the eight MEP pathway genes, including expression elements and selection markers were designed *in silico*, synthesized *in vitro* and integrated into the yeast chromosome via homologous recombination. The functionality of this pathway in *S. cerevisiae* was evaluated by the attempt to block the endogenous MVA pathway. Inhibition of the MVA pathway can be achieved using an inhibitor or by deletion of the essential genes of this pathway [26], [36], [52]–[55]. Unlike previously reported [21], chemical as well as genetic inhibition of MVA pathway revealed the non-functionality of the MEP pathway. We conclude that the previous result [21] may have derived from incomplete repression of the MVA pathway, even when higher concentrations (2 g L^−1^) of lovastatin were used, which may result from errors in activation of lovastatin by hydrolysis reducing the actual concentration of the active inhibitor, or the higher-level expression from multi-copy plasmids may have resulted in partial activation of the enzymes resulting in a functional MEP pathway. One could speculate that elevation of the number of mitochondria using galactose as a non-fermentable carbon source in the previous report [21] might have led to the functional MEP pathway. In eukaryotes, these organelles play a central role in maturation of Fe/S proteins in both mitochondria and cytosol. However, the strains presented in our work did not grow in media containing raffinose when the MVA pathway was inhibited using lovastatin (data not shown), and this strongly indicate that our previous claim of an active MEP pathway in yeast was based on lack of proper repression of the MVA pathway as also found in another study [28].

High concentrations of lovastatin could also have caused side effects which mask the operation of the MEP pathway. Thus, the inhibition of Hmg1 and Hmg2 using lovastatin has shown to result in an altered transcriptional response including the up-regulation of genes related to plasma membrane proteins, protein catabolism and ribosome biosynthesis and down-regulation of *MAF1* [56], [57] which encodes a repressor of RNA polymerase III [58]. Priviously, Kaminska and co-workers have shown an increase in tRNA levels when Maf1 is diminished [59] and consequently the demand for DMAPP, which is involved in tRNA biosynthesis, was increased [60]. Such requirement may be higher than what is provided by the MEP pathway. Gene deletion in contrast offers absolute inactivation of the MVA pathway. It has been indicated that yeast strains with deficiency in *ERG13, ERG19, ERG8*, or *ERG9* are nonviable at normal growth conditions [26], [36], [52]–[55]. Since the MEP pathway contributes to the ergosterol biosynthetic pathway through IPP and DMAPP intermediates, deletion of *ERG13, ERG19* or *ERG8* which are located upstream of these intermediates should be more efficient than using lovastatin for blocking the MVA pathway and evaluating the MEP pathway functionality. For our purpose *ERG13* is a good candidate since supplying the medium with exogenous mevalonate can complement its deletion. However, the inability of the *ERG13* deleted strain to grow showed that the MEP pathway could not complement the MVA pathway deficiency.

We hypothesize that a potential reason for the non-functionality of the MEP pathway in *S. cerevisiae* is the lack of the enzyme activity of IspG and/or IspH, which catalyze the last two reactions of the pathway. It has been reported that both enzymes, IspG and IspH, are dependent on NADPH and the flavodoxin/flavodoxin reductase redox system as electron donor for their catalytic activity [17], [18], [34], [61]–[63]. Gräwert and co-workers [32] have reported that the *in vitro* maximum activity for IspH was obtained with NADPH as co-substrate, together with recombinant flavodoxin and flavodoxin reductase from *E. coli*. Flavodoxin and flavodoxin reductase are FMN and FAD cofactor containing proteins, respectively, and it has been shown that NADPH is the preferred reducing equivalent of flavodoxin reductase compared to NADH [35]. Overexpression of flavodoxin and flavodoxin reductase might facilitate electron flux from NADPH to IspG and IspH and therefore result in increased the activity of these enzymes. A similar phenomenon was observed in biosynthesis of hydrocortisone in yeast [5]. Overexpression of the essential endogenous reductase Arh1 (adrenodoxin eeductase homolog) using a strong promoter increased the production of hydrocortisone up to 60% [5]. Both Arh1 and human ADX protein (adrenodoxin) are responsible for transferring electrons from NADPH to the related enzyme. The authors suggested that the flux of electrons was elevated as a result of *ARH1* overexpression [5]. Furthermore, both IspG and IspH are known as iron-sulfur cluster proteins which are harboring cubic type of Fe/S clusters, and it has been suggested that these [4Fe–4S] clusters participate in the electron transfer process [15]–[17], [29]–[31]. Despite the presence of Fe-S assembly machineries in yeast - the ISC system is present in the mitochondria and the CIA system is used for cytosolic FeS cluster assembly [64]–[66] - these systems may not be suitable to transfer iron-sulfur clusters to IspG and IspH. Recently, a suitable model has been proposed demonstrating the Fe/S trafficking paths leading to IspG and IspH maturation in *E. coli* [37]. The essential role of ErpA in maturation of the IspG and the IspH enzymes in *E. coli* has been indicated [33]. It was also shown that the Fe-S clusters can directly be transferred from IscU to ErpA in *E. coli* [67]. Based on these findings, the functionality of the bacterial MEP pathway was evaluated in presence of the cytosolic expression of bacterial genes *erpA*, *fpr* and *fldA* in yeast, but we still could not obtain functionality of the enzymes. Previously reported data have shown that the cytosolic localization has failed to generate a functional bacterial or human IscU while expressed in yeast [68]. Even yeast U-type homolog scaffolds (Isu1 and Isu2) playing a crucial role in maturation of both cytosolic and mitochondrial Fe-S proteins need to be expressed in mitochondria to show activity [68].

As illustrated in Figure 5, the Fe-S cluster is transferred to the last two enzymes of the MEP pathway through A-type proteins (IscA, ErpA and SufA) in three different conditions (aerobic, anaerobic and stress) [37]. A-type iron-sulfur carriers (ATCs) have initially emerged in most bacteria before being acquired by eukaryotes and a few archaea by means of horizontal gene transfer [37]. Tan and co-workers have suggested that both IscA and SufA are required for assembly of cubic Fe-S clusters in *E. coli* under aerobic condition [69]. We further constructed possible bacterial paths (aerobic and anaerobic) which are involved in transferring Fe-S clusters to IspG and IspH in the yeast cytosol (Figure 5). Co-transformation of *erpA* with either human *ISCA1* or plant derived *CpIscA* in addition to plant *ispG* and *ispH* and bacterial genes, *fpr* and *fldA* did not result in a functional MEP pathway in both aerobic and anaerobic conditions although human ISCA1 had been shown to have partial cytosolic activity in HeLa cells [38]. *S. cerevisiae* also contains two types of A-type carriers, Isa1 and Isa2, which are localized in the mitochondrial matrix and in the mitochondrial inter-membrane space, respectively [70], [71]. The mitochondrial localization is necessary for the functionality of both the Isa1 and the Isa2 protein in yeast [70]. The contribution of these proteins in the maturation process of IspG and IspH in an *E. coli* strain which has a deficiency in *erpA, iscA* and *sufA* has been demonstrated [37]. However, it has been demonstrated recently that only Isa1 can be functionally replaced by the bacterial A-type ISC proteins, ErpA, IscA and SufA [72].

In conclusion, we believe that specific physical interaction and compartmentalization would be required for *in vivo* biogenesis and transfer of essential prosthetic groups, here the iron-sulfur clusters for activation of bacterial MEP pathway enzymes in yeast. Therefore, it seems interesting to evaluate IspG and IspH expression in the mitochondria as this may represent a new interesting engineering strategy, which may even be relevant for activation of other bacterial iron-sulfur cluster proteins in yeast.

## Materials and Methods

### Strain and Plasmid Construction

Sequences of all *E. coli* MEP pathway genes including *dxs* (AAC73523), *dxr* (AAC73284), *ispD* (AAC75789), *ispE* (AAC74292), *ispF* (AAC75788), *ispG* (AAC75568), *ispH* (AAC73140) and *idi* (AAC75927) were used to construct four different integrative fragments (File S1), in which each gene was placed behind a *TEF1* or *PGK1* promoter and in front of a *CYC1* or *ADH1* terminator, respectively (Figure 2). Gene sequences were codon optimized for expression in *S. cerevisiae* and the four fragments were synthesized by DNA2.0 (Menlo Park, CA, USA). The sequences of the four fragments are presented in File S1.

The synthetic fragments were integrated into *S. cerevisiae* CEN.PK 113-13D (*MATα MAL2-8c SUC2 ura3-52*) and CEN.PK 113-1C (*MATa MAL2-8c SUC2 trp1-289 ura3-52 his3Δ1*) (kindly provided by P. Kötter, University of Frankfurt, Germany) chromosome XVI (sites YPRCΔ15 and YPRCτ3) by using a standard transformation procedure [73] and a bipartite gene targeting strategy [23]. *KanMX* as a selectable marker was looped out by methods described previously [74] and *Kluyveromyces lactis* (*K.l.*)*URA3* was looped out by selection on SD plates supplemented with 30 mg L^−1^ uracil and 750 mg L^−1^ 5-fluoroorotic acid (5-FOA), respectively. The strains harboring all MEP genes will in the following be referred to as SCISP06 and SCISP12 (Table 1).

The genes *erpA, fldA* and *fpr* were amplified by PCR using *E. coli* DH5α genomic DNA as a template and primers listed in Table 2. The *Not*I/*Sac*I restricted *fldA* fragment and *Bam*HI/*Xho*I restricted *fpr* fragment were cloned into pSP-GM1, a derivative of pSP-G1 [24], [75]. A P*_TDH3_–erpA–*T*_PGK1_* cassette was constructed by fusion PCR performed with Phusion high-fidelity DNA polymerase (Finnzymes, Espoo, Finland) and primer pair PGK1T-MreI-rev/TDH3-Kpn2I-fw, restricted by *Kpn*2I/*Mre*I and cloned into pSP-GM1 containing the *fldA* and *fpr* genes. This resulted in construction of plasmid pISP08 (Figure 3B). pISP08 was transformed into strain SCISP06 resulting in formation of strain SCISP16 (Table 1).

**Table 2:** List of oligonucleotide primers used in this study.

Primer name	Sequence
Oligonucleotide primers for verification of gene integration and transcription	
DXS up	ATGTCCTTTGATATTGCTAAATATCC
DXS down	TAGGCCAACCAAGCCTTTATC
DXR up	TGAAGCAGCTAACTATCTTGGGT
DXR down	TCGTTAAGAAGCTAGTCTCATAACTTC
ispD up	CACGACACACTTAGATGTGTGTG
ispD down	TCAAGTGTTTTCTTGGTGGATG
ispE up	ATGAGAACTCAATGGCCTTCC
ispE down	ATAACATTGCCCTATGAAGAGG
ispF up	ATGAGAATAGGTCACGGTTTCG
ispF down	TTCGTAGCCTTGATTAGCAATG
ispG up	CACAACCAAGCCCCAATACA
ispG down	TCATTTCTCCACCTGTTGGAC
ispH up	TGCAAATATTATTGGCGAATCC
ispH down	TCAATCGACCTCACGTATATCC
idi up	ATGCAGACTGAACACGTTATTCTG
idi down	TTAATTGGGTGAATGCTGACAG
URA up	GATGATGTAGTTTCTGGTTTTTAAATC
URA down	TTTAGCTTTGACATGATTAAGCTCA
KanMx up	TAGGTCTAGAGATCTGTTTAGCTTGC
KanMx down	ATTAAGGGTTCTCGAGAGCTCG
Oligonucleotide primers for gene deletions	
KanMx-1-fw	CTGAAGCTTCGTACGCTG
KanMx-1-rev	TCACCATGAGTGACGACTGA
KanMx-2-fw	TTCCAACATGGATGCTGAT
KanMx-2-rev	CTAGTGGATCTGATATCAC
ERG13-1-fw	GTTGGTGTGGTATTAAAGGA
ERG13-1-rev	CAGCGTACGAAGCTTCAG GGACTTGTCAATCAGAGTT
ERG13-2-fw	GTGATATCAGATCCACTAG CAACCTGTAAATTGGTCAC
ERG13-2-rev	CGTAAGATCTTCTAAATTTGTC
Oligonucleotide primers for verification of gene deletions	
ERG13-up-fw	TACGAGTGTGTTGAAAGTAG
ERG13-down-rev	CATTTATGAAGGGGGTTCAG
Oligonucleotide primers for amplification of the bacterial genes	
fpr-BamHI-fw	GTTGTT**GGATCC**CAGGAGAAAAACATGGCTGA
fpr -XhoI-rev	GTTGTT**CTCGAG** CGTTTATCGATAAGTAACCGCT
fldA-Not1-fw	GTTGTT**GCGGCCGC**GAGGTTATTTCACTCATGGCT
fldA-SacI-rev	GTTGTT**GAGCTC**CATCACATCAGGCATTGAGA
ErpA -fw	ATGAGTGATGACGTAGCACT
ErpA-rev	TTAGATACTAAAGGAAGAACCGCA
PGK1T-fus-fw (erpA)	TGCGGTTCTTCCTTTAGTATCTAA GGTGTTGCTTTCTTATCCGA
PGK1T-MreI-rev	GTTGTT**CGCCGGCG**GGTCGCAGAATTTTCGAGTT
TDH3-Kpn2I-fw	GTTGTT**TCCGGA**CAGTTTATCATTATCAATACTCGCC
TDH3-fus-rev (erpA)	AGTGCTACGTCATCACTCAT GAATCCGTCGAAACTAAGTTCTGGTG
Oligonucleotide primers for amplification of the human gene	
hisca-fw	ATGGCCGCCTTGACCTTGACT
hiscA-rev	TCAGATGTTGAAGGATTCACCG
PGK1T-fus-fw (iscA)	CGGTGAATCCTTCAACATCTGA GGTGTTGCTTTCTTATCCGA
TDH3-fus-rev (iscA)	AGTCAAGGTCAAGGCGGCCAT GAATCCGTCGAAACTAAGTTCTGGTG

Underlined characters correspond to flanking sequences used for fusion PCR; bold characters correspond to restriction sites.

Sequences of plant genes encoding IspG (AAN87171.1) and IspH (AAO15446.1) from *A. thaliana* were synthesized by DNA2.0 (Menlo Park, CA, USA). The sequence of *iscA* from *Homo sapiens*, *hISCA* (NP_112202.2) was synthesized by GenScript (Piscataway, NJ, USA). The sequence of *iscA* from *A. thaliana, CpIscA* (*Q9XIK3.2*) was used to construct the expression cassette, P*_TDH3_–CpIscA–*T*_PGK1_*, and this cassette was synthesized by GenScript. All synthetic genes were codon optimized for expression in *S. cerevisiae*.

The *Bam*HI/*Xho*I restricted *At-IspG* fragment and *Not*I/*Sac*I restricted *At-IspH* fragment were cloned into pSP-GM3, a derivative of the pSP-GM1 plasmid [75]. The *Pvu*II restricted fragment of pSP-GM1 including the *TEF1-PGK1* promoter region was cloned into *Pvu*II restricted pESC-HIS (Startagene,La Jolla,USA) to construct pSP-GM3. A truncated fragment of *hISCA1* encoding a protein without the mitochondrial signal peptide was amplified by PCR using the synthetic *hISCA1* as a template and primers listed in Table 2. A P*_TDH3_–hISCA1–*T*_PGK1_* cassette was constructed by fusion PCR performed with Phusion high-fidelity DNA polymerase (Finnzymes, Espoo, Finland) and primer pair PGK1T-MreI-rev/TDH3-Kpn2I-fw. Both *Kpn*2I/*Mre*I restricted P*_TDH3_–hISCA1–*T*_PGK1_* and P*_TDH3_–CpIscA–*T*_PGK1_* fragments were cloned into pSP-GM3 containing the *At-IspG* and *At-IspH* genes, respectively, resulting in pISP24 and pISP25. In the next step, pISP08 was co-transformed with either pISP24 or pISP25 (Figure 3B) into strain SCISP12 resulting in generation of strain SCISP31 and SCISP32, respectively. To create the control strain, empty plasmids, pSP-GM1 and pSP-GM3, were cloned into wild type (CEN.PK113-13D), SCISP06 and SCISP12 resulting in strains SCISP28, SCISP29 and SCISP30, respectively. The strains used in this study are listed in Table 1.

In order to delete *ERG13,* upstream and downstream flanking regions of the target gene were PCR amplified. These upstream and downstream flanking regions were fused to the 5′ and the 3′ part of the *kanMX* cassette amplified from plasmid pUG6 [74] by fusion PCR performed with Phusion high-fidelity DNA polymerase (Finnzymes). In the next step, the fused PCR fragments 1 and 2 including upstream flanking region of *ERG13*+ *loxP* - 2/3 *kanMX* and 2/3 *kanMX* - *loxP*+downstream flanking region of *ERG13*, respectively, were used for deletion by bipartite gene targeting [23]. Deletion of *ERG13* was verified by diagnostic PCR. For this purpose, PCR primers were designed to bind 400 to 500 bp up- and downstream of the start and stop codon, respectively (Table 2). All PCR products were sequenced.

### Media Composition

The transformants were selected on minimal medium plates containing 1.7 g L^−1^ yeast nitrogen base w/o amino acids and ammonium sulfate (Formedium, Hunstanton, England), 5 g L^−1^ ammonium sulfate, 0.77 g L^−1^ complete supplement mixture (CSM w/o uracil or CSM w/o uracil and histidine) (MP Biomedicals, Solon, OH, USA), 20 g L^−1^ glucose and 20 g L^−1^ agar. In media containing G418, 0.86 g L^−1^ L-glutamic acid monosodium salt monohydrate was used instead of ammonium sulfate. Filter sterilized G418 disulfide salt (Sigma-Aldrich, St. Louis, MO) was added to the media before plating to a final concentration of 200 mg L^−1^. Mevalonic acid lactone (Sigma-Aldrich) was prepared as a 500 mg L^−1^ stock solution in 2 N NaOH, incubated at 37°C for 30 min, filter sterilized, and then added to the media to reach a final concentration of 10 mg L^−1^. For anaerobic cultivations 125 µL ergosterol was added to the media from a stock solution (1 g L^−1^) that was prepared as described previously [76]. Lovastatin (Sigma) was hydrolyzed in ethanolic sodium hydroxide (15% (v/v) ethanol, 0.25% (w/v) NaOH) at 60°C for 1 h. After cooling down to room temperature, it was added to shake flasks at a final concentration of 2 g L^−1^.

### Batch Cultivation

Cotton-stopped, 50 mL Erlenmeyer flasks were used for evaluation of the MEP pathway functionality using lovastatin. The shake flasks contained 10 mL medium with the above mentioned composition. 50 mL falcon tubes containing 5 mL medium were used for seed cultures. Both seed tubes and shake flasks were incubated at 30°C and agitated in an orbital shaker at 180 rpm. Pre-cultures were used to inoculate the shake flasks to a final dry weight of 1 mg L^−1^. Cell growth was monitored by measuring the optical density at 600 nm using a Genesis20 spectrophotometer (Thermo Scientific, Madison, WI, USA). All cultivations were performed in triplicate.

## Supporting Information

File S1 **File represents DNA sequences of the four synthetic fragments which are carrying eight codon optimized bacterial genes for expression in** ***S. cerevisiae.*** For more information see text.
(PDF)
