# Supplementary material for: Reconstruction and Evaluation of the Synthetic Bacterial MEP Pathway in Saccharomyces cerevisiae
Source: PLoS One. 2012 Dec 28;7(12):e52498. doi: 10.1371/journal.pone.0052498 (PMC3532213; doi:10.1371/journal.pone.0052498)
Supplement: File S1 — File represents DNA sequences of the four synthetic fragments which are carrying eight codon optimized bacterial genes for expression in S. cerevisiae. For more information see text. (PDF) [file pone.0052498.s001.pdf]

Supplementary file:

Sequences of the four synthetic integrative cassettes including all *E. coli* MEP pathway genes which are codon optimized for efficient expression in *S. cerevisiae*.

Construct 1:

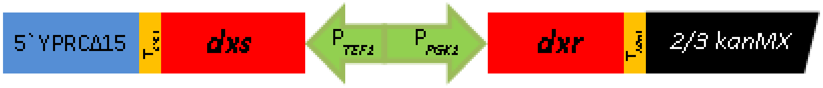

CGCCGGCGACCAGATGTTTACCTTAATTTCTTGGTGAATTAGAGAAGTACAGAAGTTTTACTATTAATCCCACCATAGAAAT
TTGTATAGGAAAGTAGTTTATTGGAGTTATTGGATATACTGTGTAAACTATTTCTTGAAATTGTAATCTTAAGATGCTCTTCT
TATTCTATTAAAAATAGAAAATGATTTTCATATTTATTTATTTATTTATATTTTGGCATTACTCTTCATCATTTTTTTCCCTCTA
AGAAGCTTCCTTTCTTTTTATAAGGATAACAAAACCAAAAGGAATATTGGGTCAGATGAATGGACGCGAATGCAAGACAG
AAGTCCAAATCACGTCAAGACAAAGAAAGAAAGAAAGAAAAACTAACACATTAATGTAGTTTTAAAATTTCAAATCCGAAC
AACAGAGCATAGGGTTTCGCAAACAAACTTAAATATATGCTAGAATAAAAATTAACTATCATCTATTGACTAGTATTCATAT
ATGACGTAATAAAATCTTCGAGCGTCCCAAAACCTTCTCAAGCAAGGTTTTCAGTATAATGTTACATGCGTACACGCGTCTG
TACAGAAAAAAAAGAAAAATTTGAAATATAAATAACGTTCTTAATACTAACATAACTATAAAAAAATAAATAGGGACCTAG
ACTTCAGGTTGTCTAACTCCTTCCTTTTCGGTTAGAGCGGA**TTTAGGCCAACCAAGCCTTTATCTTAGCTTCCATTCCAGCA**
GCGTCTAAACCAAGCTCAGCCCTCATCTCTTCTTGAGTTCCCTGTGGAATGAAAAAATCGGGTAAACCGATATTAAGAAC
AGGAACGGGCTTACGATGGGCCATCAATACTTCATTGACTCCCGAACCTGCTCCACCCATAATGGCGTTTTCTTCTACAGT
TACCAAGGCTTCGTGTGAAGCTGCCATTTCAAGGATCAAAGCTTCATCCAAGGGTTTGACAAATCTCATATCGACTAATG
TTGCATTAAGTGATTCCGCAACTTTCGCAGCTTCCGGCATTAAGGTACCAAAGTTTAGAATTGCCAATTTCTCACCTCTCC
TTTTAACTATACCTTTACCAATTGGTAGTTTTTCCAATGGGGTTAATTCCACTCCTACGGCGTTACCCCTAGGGTACCTTAC
AGCCGATGGGCCGTCATTATAATGATATCCTGTGTATAACATCTGCCTACATTCGTTTTCATCTGACGGTGTCATAATTAC
CATTTCTGGAATACATCTTAAGTACGAAAGATCAAAGGCACCTTGGTGAGTTTGACCATCAGCACCAACTATGCCGGCAC
GATCGATTGCAAACAACACAGGCAACTTTTGAATTGCTACATCATGTAGAACCTGATCATAAGCTCTTTGCAAAAAGGTT
GAATAAATGGCGACAATTGGTTTATAGCCACCGATTGCCAAACCAGCAGCAAATGTTACAGCGTGCTGCTCTGCGATGG
CAACGTCAAAATACCTGTCTGGAAACTTCCTAGAGAATTCTACCATACCAGAACCTTCTCTCATGGCAGGAGTAATTGCC
ATCAATTTATTATCCTTTGCCGCGGTTTCGCATAACCAGTCACCGAAGATTTTACTATAAGATGGCAAACCACCAGATGA
CTTCGGTAAGCATCCAGAGCTTGGATCGAATTTGGGAACGGCGTGAAAGGTAATCGGATCTTTTTCAGCCGGCTCATAA
CCACGACCCTTCTTAGTCATGATATGTAGGAATTGTGGGCCCTTCAGATCTCTCATGTTTTTCAAAGTTGTGATCAATCCT
AATACATCGTGCCCATCCACCGGTCCTATGTAATTGAAACCCAACTCCTCAAACAATGTACCTGGAACCACCATACCCTTT
ATATGTTCCTCCGTCCTTTTTAACAACTCCTTGATTGGTGGAACCCCGCTGAAAACTTTCTTACCTCCTTCCCTTAGGGACG
AGTATAGTTTACCGCTTAGTAATTGAGCCAAATGATTATTCAGGGCACCAACGTTCTCTGAAATCGACATCTCATTATCGT
TTAAAATTACCAACATGTCAGGTCTTATGTCTCCTGCATGGTTCATTGCTTCAAAAGCCATACCAGCGGTTATGGCACCGT
CGCCAATAACACAGACAGTTCTCCTATTCTTGCCTTCCTTTTCGGCCGCGACCGCGATACCAATACCAGCACTAATTGAGG
TACTGCTATGTCCGACGCTTAACACATCATATTCACTTTCGCCCCTCCACGGAAAAGGATGTAAGCCTCCCTTTTGACGAA
TAGTTCCAATTTTATCTCTCCTACCTGTTAGAATTTTATGAGGATAAGCTTGGTGACCCACATCCCAAATTAGTTGGTCAA
AGGGCGTGTTGTAGACATAGTGAAGTGCTACAGTTAGTTCCACAGTTCCCAATCCCGACGCAAAGTGGCCAGATGATCT
CGAAACAGAGTCCAACAGATACCTTCTTAACTCATCACACAACTTAGGCAAACTTTCTTTAGGCAATAAACGTAATTCTT
GAGTACTATCTACTAATGCCAATGTTGGATATTTAGCAATATCAAAGGACATGGATCCTTGTAATTAAAACTTAGATTAG
ATTGCTATGCTTTCTTTCTAATGAGCAAGAAGTAAAAAAAGTTGTAATAGAACAAGAAAAATGAAACTGAAACTTGAGAAA
TTGAAGACCGTTTATTAACTTAAATATCAATGGGAGGTCATCGAAAGAGAAAAAAATCAAAAAAAAAAATTTTCAAGAAA
AAGAAACGTGATAAAAATTTTTATTGCCTTTTTCGACGAAGAAAAAGAAACGAGGCGGTCTCTTTTTTCTTTTCCAAACCTT
TAGTACGGGTAATTAACGACACCCTAGAGGAAGAAAGAGGGGAAATTTAGTATGCTGTGCTTGGGTGTTTTGAAGTGGTA
CGGCGATGCGCGGAGTCCGAGAAAATCTGGAAGAGTAAAAAAGGAGTAGAAACATTTTGAAGCTATGGTGTGTGCGGCC
GGCCGGAAGTACCTTCAAAGAATGGGGTCTTATCTTGTTTTGCAAGTACCACTGAGCAGGATAATAATAGAAATGATAATA

TACTATAGTAGAGATAACGTCGATGACTTCCCATACTGTAATTGCTTTTAGTTGTGTATTTTTAGTGTGCAAGTTTCTGTAAA
TCGATTAATTTTTTTTTCTTTCCTCTTTTTATTAACCTTAATTTTTATTTTAGATTCCTGACTTCAACTCAAGACGCACAGATAT
TATAACATCTGCATAATAGGCATTTGCAAGAATTACTCGTGAGTAAGGAAAGAGTGAGGAACTATCGCATACCTGCATTTA
AAGATGCCGATTTGGGCGCGAATCCTTTATTTTGGCTTCACCCTCATACTATTATCAGGGCCAGAAAAAGGAAGTGTTTCCC
TCCTTCTTGAATTGATGTTACCCTCATAAAGCACGTGGCCTCTTATCGAGAAAGAAATTACCGTCGCTCGTGATTTGTTTGC
AAAAAGAACAAAACTGAAAAAACCCAGACACGCTCGACTTCCTGTCTTCCTATTGATTGCAGCTTCCAATTTCGTCACACAA
CAAGGTCCTAGCGACGGCTCACAGGTTTTGTAACAAGCAATCGAAGGTTCTGGAATGGCGGGAAAGGGTTTAGTACCACA
TGCTATGATGCCCACTGTGATCTCCAGAGCAAAGTTCGTTCGATCGTACTGTTACTCTCTCTCTTTCAAACAGAATTGTCCGA
ATCGTGTGACAACAACAGCCTGTTCTCACACACTCTTTTCTTCTAACCAAGGGGGTGGTTTAGTTTAGTAGAACCTCGTGAA
ACTTACATTTACATATATATAAACTTGCATAAATTGGTCAATGCAAGAAATACATATTTGGTCTTTTCTAATTCGTAGTTTTTC
AAGTTCTTAGATGCTTTCTTTTTCTCTTTTTTACAGATCATCAAGGAAGTAATTATCTACTTTTTACAACAAATATAAAACAAG
CGGCCGC**ATGAAGCAGCTAACTATCTTGGGTTCGACTGGTTCTATCGGTTGCTCAACTCTTGATGTAGTAAGGCACAATC**
CAGAACATTTCAGAGTAGTCGCTCTAGTAGCCGGTAAAAATGTTACGAGAATGGTTGAACAATGTTTGGAATTCTCTCCT
CGTTACGCTGTTATGGATGATGAAGCCTCGGCAAAGTTACTGAAGACGATGTTACAACAACAAGGGAGTAGAACTGAA
GTCCTGTCTGGACAGCAGGCAGCTTGTGACATGGCAGCCCTTGAAGACGTCGATCAGGTTATGGCAGCAATTGTAGGTG
CCGCTGGTCTTCTTCCGACATTGGCGGCTATTAGAGCCGGTAAAACAATTTTACTTGCTAATAAGGAATCTCTTGTAACTT
GTGGAAGATTGTTTATGGACGCTGTAAAACAGTCTAAAGCTCAATTGCTACCTGTCGACTCTGAACATAACGCAATATTT
CAATCTCTACCACAGCCTATTCAACACAATTTGGGTTACGCGGACTTAGAGCAGAACGGAGTAGTAAGTATCCTGTTGAC
AGGAAGCGGAGGACCATTTAGAGAGACTCCATTGAGAGACTTAGCCACCATGACCCCAGATCAAGCATGTCGTCATCCG
AATTGGTCCATGGGTCGTAAGATATCTGTTGACTCAGCCACAATGATGAATAAAGGTCTGGAGTATATCGAAGCTAGAT
GGCTATTTAATGCCTCAGCTTCCCAGATGGAAGTGCTAATTCATCCGCAAAGCGTCATTCATTCCATGGTCAGATATCAG
GATGGCTCAGTCTTAGCGCAATTGGGTGAACCAGATATGAGAACACCAATTGCCCATACTATGGCCTGGCCGAATAGGG
TCAATTCAGGAGTAAAGCCTCTAGACTTTTGCAAATTATCAGCACTGACGTTTGCTGCTCCCGATTACGACAGGTACCCTT
GCCTGAAGCTTGCCATGGAGGCTTTTGAACAAGGCCAGGCAGCTACAACCGCACTAAACGCCGCTAACGAAATTACTGT
TGCTGCTTTCCTTGCTCAACAGATTCGTTTTACCGACATTGCAGCACTGAACTTATCAGTTTTAGAAAAGATGGACATGA
GGGAGCCACAGTGTGTCGACGATGTTCTTTCCGTAGACGCTAATGCTAGGGAAGTTGCTAGAAAAGAAGTTATGAGAC
TAGCTTCTTAACGAATTTCTTATGATTTATGATTTTTATTATTAAATAAGTTATAAAAAAAAATAAGTGTATACAAATTTTAAA
GTGACTCTTAGGTTTTAAAACGAAAATTCTTATTCTTGAGTAACTCTTTCCTGTAGGTCAGGTTGCTTTCTCAGGTATAGCAT
GAGGTCGCTCATAACTTCGTATAATGTATGCTATACGAAGTTATTAGGTCTAGAGATCTGTTTAGCTTGCCTCGTCCCCGCC
GGGTCACCCGGCCAGCGACATGGAGGCCCAGAATACCCTCCTTGACAGTCTTGACGTGCGCAGCTCAGGGGCATGATGTG
ACTGTCGCCCGTACATTTAGCCCATACATCCCCATGTATAATCATTTGCATCCATACATTTTGATGGCCGCACGGCGCGAAG
CAAAAATTACGGCTCCTCGCTGCAGACCTGCGAGCAGGGAAACGCTCCCCTCACAGACGCGTTGAATTGTCCCCACGCCGC
GCCCCTGTAGAGAAATATAAAAGGTTAGGATTTGCCACTGAGGTTCTTCTTTCATATACTTCCTTTTAAAATCTTGCTAGGA
TACAGTTCTCACATCACATCCGAACATAAACAACCATGGGTAAGGAAAAGACTCACGTTTCGAGGCCGCGATTAAATTCCA
ACATGGATGCTGATTTATATGGGTATAAATGGGCTCGCGATAATGTCGGGCAATCAGGTGCGACAATCTATCGATTGTATG
GGAAGCCCGATGCGCCAGAGTTGTTTCTGAAACATGGCAAAGGTAGCGTTGCCAATGATGTTACAGATGAGATGGTCAGA
CTAAACTGGCTGACGGAATTTATGCCTCTTCCGACCATCAAGCATTTTATCCGTACTCCTGATGATGCATGGTTACTCACCA
CTGCGATCCCCGGCAAAACAGCATTCCAGGTATTAGAAGAATATCCTGATTCAGGTGAAAATATTGTTGATGCGCTGGCAG
TGTTCCTGCGCCGGTTGCATTCGATTCCTGTTTGTAATTGTCCTTTTAACAGCGATCGCGTATTTCGTCTCGCTCAGGCGCAA
TCACGAATGAATAACGGTTTGGTTGATGCGAGTGATTTTGATGACGAGCGTAATGGCTGGCCTGTTGAACAAGTCTGGAA
AGAAATGCATAAGCTTTTGCCATTCTCACCGGATTCAGTCGTCACTCATGGTGATTTCTCACTTGATAACCTTATTTTTGACG
AGGGGAAATTAATAGGTTGTAGGCGCGCC

Construct 2:

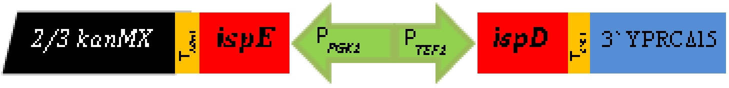

GCCGGCGGAAGAATATCCTGATTCAGGTGAAAATATTGTTGATGCGCTGGCAGTGTTCCTGCGCCGGTTGCATTCGATTCC
TGTTTGTAATTGTCCTTTTAACAGCGATCGCGTATTTCGTCTCGCTCAGGCGCAATCACGAATGAATAACGGTTTGGTTGAT
GCGAGTGATTTTGATGACGAGCGTAATGGCTGGCCTGTTGAACAAGTCTGGAAAGAAATGCATAAGCTTTTGCCATTCTCA
CCGGATTCAGTCGTCACTCATGGTGATTTCTCACTTGATAACCTTATTTTTGACGAGGGGAAATTAATAGGTTGTATTGATG
TTGGACGAGTCGGAATCGCAGACCGATACCAGGATCTTGCCATCCTATGGAACTGCCTCGGTGAGTTTTCTCCTTCATTACA
GAAACGGCTTTTTCAAAAATATGGTATTGATAATCCTGATATGAATAAATTGCAGTTTCATTTGATGCTCGATGAGTTTTTCT
AATCAGTACTGACAATAAAAAGATTCTTGTTTTCAAGAACTTGTCATTTGTATAGTTTTTTTATATTGTAGTTGTTCTATTTTA
ATCAAATGTTAGCGTGATTTATATTTTTTTTCGCCTCGACATCATCTGCCCAGATGCGAAGTTAAGTGCGCAGAAAGTAATA
TCATGCGTCAATCGTATGTGAATGCTGGTCGCTATACTGCTGTCGATTCGATACTAACGCCGCCATCCAGTGTCGAAAACG
AGCTCTCGAGAACCCTTAATATAACTTCGTATAATGTATGCTATACGAAGTTATGAGCGACCTCATGCTATACCTGAGAAAG
CAACCTGACCTACAGGAAAGAGTTACTCAAGAATAAGAATTTTCGTTTTAAAACCTAAGAGTCACTTTAAAATTTGTATACA
CTTATTTTTTTTATAACTTATTTAATAATAAAAATCATAAATCATAAGAAATTCG**TTATAACATTGCCCTATGAAGAGGGGA**
TAGGTTTGCACCTTTAGCAACGAATCCATTCAACCACTCAGGTGCTTGCTCTAACACTTGTCTAGCTTCAGATTCTGTGTC
AAATTCGGCAAATACACAGGCTCCTGTACCGGTAAGTCTACTTGGAGCGTACTCCAGAAGCCACGATAAAACGGCATCA
ACTTCACGAAACCTTTTTCTTGCAATTACTTCACAGTCATTACTAAATTCGCATTTTAACAGAGTTTCAATAGAACGTTTTG
GGGTGTTTCTCGGCAACTCCGGGTCTTTGAATATAACTGGCGTTGGAATCGAGACGCCTGGATGCGCAACTAGATACCA
TTTCTCTGGAGGATCTACTGGAGTAAGTATCTCTCCGACACCTTCTGCGAATGCCGCGTGACCACGAACGAAAACAGGA
ACATCAGCCCCCAATGTTAAACCCATTTCAGCTAACTCGTCCATTGACAGTCCGCACTGCCACAAGTGATTTAGGGCGAC
TAAAACGGTAGCTGCATTAGATGACCCTCCACCAAGTCCACCACCCATCGGCAACCTCTTATCTATAGATATATTGGCACC
TGAACCAGTTGGCAATCTGCCGCTATCGGCTGCGGTCTTCATTAATAGCCTTGCGGCCCTAACAATCAAATTGTCTTCATG
TTCGACGCCTTCAACAGGTGTAAGCAACCTTATATCCCCATCATCTCTCAACTCTATTGATATAGTATCGCCATAATCTAG
GAACTGGAATAAAGTCTGTAGGGTATGGTAACCATCAGCTCTTTGACCAGTAATGTACAAGAACAAGTTCAGTTTAGCT
GGGGAAGGCCATTGAGTTCTCATGCGGCCGCTTGTTTTATATTTGTTGTAAAAAGTAGATAATTACTTCCTTGATGATCTGT
AAAAAAGAGAAAAAGAAAGCATCTAAGAACTTGAAAAACTACGAATTAGAAAAGACCAAATATGTATTTCTTGCATTGAC
CAATTTATGCAAGTTTATATATATGTAAATGTAAGTTTCACGAGGTTCTACTAAACTAAACCACCCCCTTGGTTAGAAGAAA
AGAGTGTGTGAGAACAGGCTGTTGTTGTCACACGATTCGGACAATTCTGTTTGAAAGAGAGAGAGTAACAGTACGATCGA
ACGAACTTTGCTCTGGAGATCACAGTGGGCATCATAGCATGTGGTACTAAACCCTTTCCCGCCATTCCAGAACCTTCGATTG
CTTGTTACAAAACCTGTGAGCCGTCGCTAGGACCTTGTTGTGTGACGAAATTGGAAGCTGCAATCAATAGGAAGACAGGA
AGTCGAGCGTGTCTGGGTTTTTTCAGTTTTGTTCTTTTTGCAAACAAATCACGAGCGACGGTAATTTCTTTCTCGATAAGAG
GCCACGTGCTTTATGAGGGTAACATCAATTCAAGAAGGAGGGAAACACTTCCTTTTTCTGGCCCTGATAATAGTATGAGGG
TGAAGCCAAAATAAAGGATTCGCGCCCAAATCGGCATCTTTAAATGCAGGTATGCGATAGTTCCTCACTCTTTCCTTACTCA
CGAGTAATTCTTGCAAATGCCTATTATGCAGATGTTATAATATCTGTGCGTCTTGAGTTGAAGTCAGGAATCTAAAATAAAA
ATTAAGGTTAATAAAAAGAGGAAAGAAAAAAAAAATTAATCGATTTACAGAAACTTGCACACTAAAAATACACAACTAAAA
GCAATTACAGTATGGGAAGTCATCGACGTTATCTCTACTATAGTATATTATCATTTCTATTATTATCCTGCTCAGTGGTACTT
GCAAAACAAGATAAGACCCCATTCTTTGAAGGTACTTCCGGCCGGCCGCACACACCATAGCTTCAAAATGTTTCTACTCCTT
TTTTACTCTTCCAGATTTTCTCGGACTCCGCGCATCGCCGTACCACTTCAAAACACCCAAGCACAGCATACTAAATTTCCCCT
CTTTCTTCCTCTAGGGTGTCGTTAATTACCCGTACTAAAGGTTTGGAAAAGAAAAAAGAGACCGCCTCGTTTCTTTTTCTTC
GTCGAAAAAGGCAATAAAAATTTTTATCACGTTTCTTTTTCTTGAAAATTTTTTTTTTTGATTTTTTTCTCTTTCGATGACCTCC
CATTGATATTTAAGTTAATAAACGGTCTTCAATTTCTCAAGTTTCAGTTTCATTTTTCTTGTTCTATTACAACTTTTTTTACTTC
TTGCTCATTAGAAAGAAAGCATAGCAATCTAATCTAAGTTTTAATTACAAGGATCC**ATGGCCACGACACACTTAGATGTGT**
GTGCCGTAGTCCCAGCAGCCGGCTTCGGTAGAAGAATGCAGACCGAATGTCCTAAACAGTACCTATCTATCGGTAACCA
AACCATTTTAGAACATAGTGTACACGCATTATTAGCGCACCCGAGGGTGAAAAGAGTTGTTATCGCCATCTCGCCAGGG
GATAGCCGTTTTGCTCAATTGCCTTTAGCCAACCATCCTCAAATCACCGTGGTAGATGGTGGTGATGAACGTGCTGATTC
CGTCCTTGCAGGTTTAAAAGCGGCGGGAGATGCTCAATGGGTTCTGGTTCATGACGCAGCCAGACCATGTCTACATCAG
GATGACCTGGCCAGACTGTTGGCACTTAGTGAAACTTCTAGGACGGGAGGTATTCTTGCAGCTCCTGTCAGAGATACCA

**TGAAGAGAGCCGAACCTGGCAAGAATGCAATAGCTCATACAGTTGATAGAAACGGTTTGTGGCACGCTTTAACTCCACA
ATTTTTTCCTAGAGAACTATTGCACGATTGTCTTACGAGGGCACTAAATGAGGGCGCAACCATAACCGATGAAGCTTCCG
CTCTTGAATACTGTGGTTTCCATCCACAGCTTGTAGAGGGTAGAGCAGATAACATAAAAGTCACTCGTCCAGAGGATCT
AGCTTTGGCAGAATTTTACTTGACACGTACCATCCACCAAGAAAACACTTGA**ATCCGCTCTAACCGAAAAGGAAGGAGTT
AGACAACCTGAAGTCTAGGTCCCTATTTATTTTTTTATAGTTATGTTAGTATTAAGAACGTTATTTATATTTCAAATTTTTCTT
TTTTTTCTGTACAGACGCGTGTACGCATGTAACATTATACTGAAAACCTTGCTTGAGAAGGTTTTGGGACGCTCGAAGAAT
AAATTCGTTAAATAACGGTGTGTTGAAATGTTTACCGTAACTTGTAACAGCTCTAACAACTCATACCTGCTATGTACTGATT
CCAAGAAAAAAAAAAATGGAAGGTCGGGATGAGCATATACAAGCACTAAGAAGAACAATACAGAACTCTACACGGTATTAT
TGTGCTACAAGCTCGAGTAAAACCGAGTGTTTTGACGATACTAACGTTGTTAAGAAAGTAACTTGTTATCAAACTCATTACC
AACTTGTGATTAATTGGTGAATAATATGATAATTGTCGAAATTCCATTGTTGGTAAAGCCTATAATATTATGTATACAGATT
ATACTAGAAATTCTCTCGAGAATATAAGAATCCCCAAAATTGAATCGGTATTTCTACATACTAATATTACCATTACTTCTCCT
TTCGTTTTATATGTTTCATTCCTATTACATTATCGATCTTTGCATTTCAGCTTCCATTATATTTGATGTCTGTTTTATGTCCCCA
CGTGGCGCGCC

Construct 3:

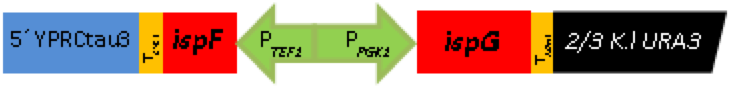

CGCCGGCGATGTTACCATAATGTTCTTGTCCATTTTGCATACACTTTAAATATTCATTTGATTTCTCAGGGTTCATGATCATA
ATAAATTGCGCATTCGCAAGGCGGTAGTATTATAATGGGGTCCATCATTCTGTAGCAAGAAGTTACAGTACGCTGTTCAAG
CGTTAAACAAGATAAGTAATCTCGAATGAAACATTCATATTTCGCATGAGCCAACATACAGTTGCTGAGTAATCTTCATTGC
GCTTATTTATCGGCATTGAGATTGTAAAGGAAGTAAAACGCATTTTTGCAGATCTGTTCTCTTATGTATTTTTAATCGTCCTT
GTATGGAAGTATCAAAGGGGACGTTCTTCACCTCCTTGGAATGTGTTCCACTATCCTGTACATGTGAAATGTATAAAAGCC
AAAATTGACCTTGTCATTACCAATGAATGCTGTTTTTGCAGAAATAACGAGATATCTGCAATAAAAGCAAAAGTAAGTTTG
ATAGCAAGAGGTTGTTGCTTCGAGCGTCCCAAAACCTTCTCAAGCAAGGTTTTCAGTATAATGTTACATGCGTACACGCGT
CTGTACAGAAAAAAAAGAAAAATTTGAAATATAAATAACGTTCTTAATACTAACATAACTATAAAAAAATAAATAGGGACC
TAGACTTCAGGTTGTCTAACTCCTTCCTTTTCGGTTAGAGCGGA**TCTATTTCGTAGCCTTGATTAGCAATGCAACTGCTTCG**
CAAGCAATACCCTCTCCTCTACCAGTGAAACCCAACTTTTCAGTAGTGGTTGCTTTCACATTAACATCGTCCATGTGACAA
CCCAAGTCTTCTGCAATAAAAACACGCATTTGTGGTATATGAGGCAACATTTTGGGAGCTTGAGCTATAATCGTAACATC
CACATTACCTAAGGTGTAACCTTTGGCCTGAATTCTTCTCCAGGCTTCCCTTAACAATTCTCTGGAATCAGCACCTTTGAA
AGCTGGGTCAGTATCTGGGAATAACTTTCCAATGTCGCCCAAGGCTGCTGCCCCCAACAATGCGTCGGTTAAAGCATGA
AGAGCGACATCACCATCAGAGTGGGCTAATAACCCTTTTTCATACGGTATTCTCACGCCCCCGATGATAATGGGCCCTTC
TCCTCCAAAAGCATGTACATCGAAACCGTGACCTATTCTCATGGATCCTTGTAATTAAAACTTAGATTAGATTGCTATGCTT
TCTTTCTAATGAGCAAGAAGTAAAAAAAGTTGTAATAGAACAAGAAAAATGAAACTGAAACTTGAGAAATTGAAGACCGT
TTATTAACTTAAATATCAATGGGAGGTCATCGAAAGAGAAAAAAATCAAAAAAAAAAATTTTCAAGAAAAAGAAACGTGA
TAAAAATTTTTATTGCCTTTTTCGACGAAGAAAAAGAAACGAGGCGGTCTCTTTTTTCTTTTCCAAACCTTTAGTACGGGTA
ATTAACGACACCCTAGAGGAAGAAAGAGGGGAAATTTAGTATGCTGTGCTTGGGTGTTTTGAAGTGGTACGGCGATGCG
CGGAGTCCGAGAAAATCTGGAAGAGTAAAAAAGGAGTAGAAACATTTTGAAGCTATGGTGTGTGCGGCCGGCCGGAAGT
ACCTTCAAAGAATGGGGTCTTATCTTGTTTTGCAAGTACCACTGAGCAGGATAATAATAGAAATGATAATATACTATAGTA
GAGATAACGTCGATGACTTCCCATACTGTAATTGCTTTTAGTTGTGTATTTTTAGTGTGCAAGTTTCTGTAAATCGATTAATT
TTTTTTTCTTTCCTCTTTTTATTAACCTTAATTTTTATTTTAGATTCCTGACTTCAACTCAAGACGCACAGATATTATAACATCT
GCATAATAGGCATTTGCAAGAATTACTCGTGAGTAAGGAAAGAGTGAGGAACTATCGCATACCTGCATTTAAAGATGCCG
ATTTGGGCGCGAATCCTTTATTTTGGCTTCACCCTCATACTATTATCAGGGCCAGAAAAAGGAAGTGTTTCCCTCCTTCTTG
AATTGATGTTACCCTCATAAAGCACGTGGCCTCTTATCGAGAAAGAAATTACCGTCGCTCGTGATTTGTTTGCAAAAAGAA
CAAAACTGAAAAAACCCAGACACGCTCGACTTCCTGTCTTCCTATTGATTGCAGCTTCCAATTTCGTCACACAACAAGGTCC
TAGCGACGGCTCACAGGTTTTGTAACAAGCAATCGAAGGTTCTGGAATGGCGGGAAAGGGTTTAGTACCACATGCTATGA
TGCCCACTGTGATCTCCAGAGCAAAGTTCGTTCGATCGTACTGTTACTCTCTCTCTTTCAAACAGAATTGTCCGAATCGTGT
GACAACAACAGCCTGTTCTCACACACTCTTTTCTTCTAACCAAGGGGGTGGTTTAGTTTAGTAGAACCTCGTGAAACTTACA
TTTACATATATATAAACTTGCATAAATTGGTCAATGCAAGAAATACATATTTGGTCTTTTCTAATTCGTAGTTTTTCAAGTTCT
TAGATGCTTTCTTTTTCTCTTTTTTACAGATCATCAAGGAAGTAATTATCTACTTTTTACAACAAATATAAAACAAGCGGCCG
C**ATGCACAACCAAGCCCCAATACAAAGGAGAAAATCAACTAGAATATACGTTGGGAATGTTCCCATAGGCGATGGAGC**
GCCGATTGCCGTACAGAGTATGACCAATACGAGGACTACAGACGTAGAAGCTACTGTCAATCAAATCAAGGCGTTAGA
AAGAGTCGGGGCAGACATCGTTAGAGTTAGTGTCCCTACAATGGATGCAGCAGAAGCATTTAAGCTAATAAAACAGCA
AGTTAACGTACCATTGGTGGCAGATATTCACTTTGACTATAGAATCGCGTTAAAAGTGGCAGAGTATGGTGTTGATTGC
CTAAGGATCAACCCAGGAAACATTGGGAACGAGGAAAGGATCAGGATGGTCGTAGACTGTGCAAGGGATAAGAACAT
CCCTATCAGAATTGGCGTGAATGCGGGATCGCTGGAAAAAGACTTGCAGGAAAAATATGGCGAACCAACTCCGCAAGC
ATTGTTGGAAAGCGCTATGAGACATGTAGATCATTTGGACAGGCTTAATTTTGATCAATTTAAAGTAAGCGTGAAGGCT
TCGGACGTATTTTTGGCTGTGGAGTCTTATAGATTGTTAGCCAAGCAGATTGATCAACCATTACATCTGGGCATTACCGA
AGCTGGCGGTGCTAGATCCGGTGCTGTAAAGTCAGCAATTGGGCTGGGTCTTTTACTATCGGAAGGTATTGGCGATACT
CTGAGAGTTAGCCTTGCCGCTGATCCTGTCGAAGAAATCAAGGTAGGTTTTGATATACTAAAATCTTTAAGGATACGTA
GTCGTGGAATCAACTTTATTGCTTGTCCCACATGTTCGAGACAAGAATTTGATGTAATTGGTACTGTAAACGCGTTGGAA
CAAAGATTGGAAGACATTATTACACCAATGGACGTGTCTATTATAGGTTGTGTTGTAAACGGGCCTGGGGAAGCTTTAG
TCTCAACATTAGGTGTTACTGGGGGTAACAAGAAGAGTGGTCTATATGAAGATGGAGTCAGAAAGGATCGTTTAGATA

ATAATGATATGATAGACCAATTGGAGGCAAGAATCCGTGCGAAAGCATCACAATTGGATGAGGCACGTAGAATAGATG
TCCAACAGGTGGAGAAATGACGAATTTCTTATGATTTATGATTTTTATTATTAAATAAGTTATAAAAAAAATAAGTGTATAC
AAATTTTAAAGTGACTCTTAGGTTTTAAAACGAAAATTCTTATTCTTGAGTAACTCTTTCCTGTAGGTCAGGTTGCTTTCTCA
GGTATAGCATGAGGTCGCTCGTGATTCTGGGTAGAAGATCGGTCTGCATTGGATGGTGGTAACGCATTTTTTTACACACAT
TACTTGCCTCGAGCATCAAATGGTGGTTATTCGTGGATCTATATCACGTGATTTGCTTAAGAATTGTCGTTCATGGTGACAC
TTTTAGCTTTGACATGATTAAGCTCATCTCAATTGATGTTATCTAAAGTCATTTCAACTATCTAAGATGTGGTTGTGATTGGG
CCATTTTGTGAAAGCCAGTACGCCAGCGTCAATACACTCCCGTCAATTAGTTGCACCATGTCCACAAAATCATATACCAGTA
GAGCTGAGACTCATGCAAGTCCGGTTGCATCGAAACTTTTACGTTTAATGGATGAAAAGAAGACCAATTTGTGTGCTTCTC
TTGACGTTCGTTCGACTGATGAGCTATTGAAACTTGTTGAAACGTTGGGTCCATACATTTGCCTTTTGAAAACACACGTTGA
TATCTTGGATGATTTCAGTTATGAGGGTACTGTCGTTCCATTGAAAGCATTGGCAGAGAAATACAAGTTCTTGATATTTGA
GGACAGAAAATTCGCCGATATCGGTAACACAGTCAAATTACAATATACATCGGGCGTTTACCGTATCGCAGAATGGTCTGA
TATCACCAACGCCCACGGGGTTACTGGTGCTGGTATTGTTGCTGGCTTGAAACAAGGTGCGCAAGAGGTCACCAAAGAAC
CAAGGGGATTATTGATGCTTGCTGAATTGTCTTCCAAGGGTTCTCTAGCACACGGTGAATATACTAAGGGTACCGTTGATA
TTGCAAAGAGTGATAAAGATTTCGTTATTGGGTTCATTGCTCGGCGCGC

Construct 4:

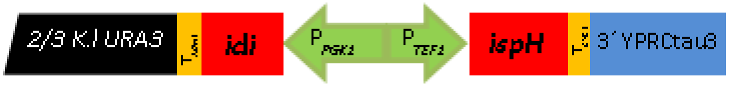

CGCCGGCGTATTTGAGGACAGAAAATTCGCCGATATCGGTAACACAGTCAAATTACAATATACATCGGGCGTTTACCGTAT
CGCAGAATGGTCTGATATCACCAACGCCCACGGGGTTACTGGTGCTGGTATTGTTGCTGGCTTGAAACAAGGTGCGCAAG
AGGTCACCAAAGAACCAAGGGGATTATTGATGCTTGCTGAATTGTCTTCCAAGGGTTCTCTAGCACACGGTGAATATACTA
AGGGTACCGTTGATATTGCAAAGAGTGATAAAGATTTCGTTATTGGGTTCATTGCTCAGAACGATATGGGAGGAAGAGAA
GAAGGGTTTGATTGGCTAATCATGACCCCAGGTGTAGGTTTAGACGACAAAGGCGATGCATTGGGTCAGCAGTACAGAAC
CGTCGACGAAGTTGTAAGTGGTGGATCAGATATCATCATTGTTGGCAGAGGACTTTTCGCCAAGGGTAGAGATCCTAAGG
TTGAAGGTGAAAGATACAGAAATGCTGGATGGGAAGCGTACCAAAAGAGAATCAGCGCTCCCCATTAATTATACAGGAA
ACTTAATAGAACAAATCACATATTTAATCTAATAGCCACCTGCATTGGCACGGTGCAACACTACTTCAACTTCATCTTACAA
AAAGATCACGTGATCTGTTGTATTGAACTGAAAATTTTTTGTTTGCTTCTCTCTCTCTCTCTTTCATTATGTGAGATTTAAAAA
CCAGAAACTACATCATCGTGATTCTGGGTAGAAGATCGGTCTGCATTGGATGGTGGTAACGCATTTTTTTACACACATTACT
TGCCTCGAGCATCAAATGGTGGTTATTCGTGGATCTATATCACGTGATTTGCTTAAGAATTGTCGTTCATGGTGACACGAG
CGACCTCATGCTATACCTGAGAAAGCAACCTGACCTACAGGAAAGAGTTACTCAAGAATAAGAATTTTCGTTTTAAAACCT
AAGAGTCACTTTAAAATTTGTATACACTTATTTTTTTTATAACTTATTTAATAATAAAAATCATAAATCATAAGAAATTCG**CT**
ATTTTAATTGGGTGAATGCTGACAGCCTTTTTCTTGCCTCTCTATTCGTGGCCTGCATCACCATCCATGGAGAAAATGCCC
AAGGCGTTGCATCTATACCATGCAAAACATCAGCCAAGTCACACCATTGATAATCCATAACCTCATCGTCATTAATCTGC
AAGGCCGATGTCGTACGTGCAGCAAACACCGGACACACTTCGTTTTCGACTATACCACTGGGATCTGTCGCTCTGTATCT
AAAGTCTGGATATATAGATTCAGGAGGTGTAATTTCGACTCCTAGTTCGTATCTGCACCTCCTGATCACGGCATCCTCATT
TGACTCGCCCAATTGTGGATGCCCACATACACTATTAGTCCAAACTCCAGGCCAAGCCTTCTTCGACAAGGCTCTTCTTGT
AACTAGAAGCTGGCCTTTAGCGTTAAAAAGCCAAGACGAGAATGCCAAATGTAATCTTGTGTCAGCTGTATGAGCAGCG
TACTTTTCAAGAGTTCCAGTAGGGACACCTTGAGCGTTTAACAGAATAACGTGTTCAGTCTGCATGCGGCCGCTTGTTTT
ATATTTGTTGTAAAAAGTAGATAATTACTTCCTTGATGATCTGTAAAAAAGAGAAAAAGAAAGCATCTAAGAACTTGAAAA
ACTACGAATTAGAAAAGACCAAATATGTATTTCTTGCATTGACCAATTTATGCAAGTTTATATATATGTAAATGTAAGTTTC
ACGAGGTTCTACTAAACTAAACCACCCCCTTGGTTAGAAGAAAAGAGTGTGTGAGAACAGGCTGTTGTTGTCACACGATTC
GGACAATTCTGTTTGAAAGAGAGAGAGTAACAGTACGATCGAACGAACTTTGCTCTGGAGATCACAGTGGGCATCATAGC
ATGTGGTACTAAACCCTTTCCCGCCATTCCAGAACCTTCGATTGCTTGTTACAAAACCTGTGAGCCGTCGCTAGGACCTTGT
TGTGTGACGAAATTGGAAGCTGCAATCAATAGGAAGACAGGAAGTCGAGCGTGTCTGGGTTTTTTCAGTTTTGTTCTTTTT
GCAAACAAATCACGAGCGACGGTAATTTCTTTCTCGATAAGAGGCCACGTGCTTTATGAGGGTAACATCAATTCAAGAAG
GAGGGAAACACTTCCTTTTTCTGGCCCTGATAATAGTATGAGGGTGAAGCCAAAATAAAGGATTCGCGCCCAAATCGGCA
TCTTTAAATGCAGGTATGCGATAGTTCCTCACTCTTTCCTTACTCACGAGTAATTCTTGCAAATGCCTATTATGCAGATGTTA
TAATATCTGTGCGTCTTGAGTTGAAGTCAGGAATCTAAAATAAAAATTAAGGTTAATAAAAAGAGGAAAGAAAAAAAAAT
TAATCGATTTACAGAAACTTGCACACTAAAAATACACAACTAAAAGCAATTACAGTATGGGAAGTCATCGACGTTATCTCT
ACTATAGTATATTATCATTTCTATTATTATCCTGCTCAGTGGTACTTGCAAAACAAGATAAGACCCCATTCTTTGAAGGTACT
TCCGGCCGGCCGCACACACCATAGCTTCAAAATGTTTCTACTCCTTTTTTACTCTTCCAGATTTTCTCGGACTCCGCGCATCG
CCGTACCACTTCAAAACACCCAAGCACAGCATACTAAATTTCCCCTCTTTCTTCCTCTAGGGTGTCGTTAATTACCCGTACTA
AAGGTTTGGAAAAGAAAAAAGAGACCGCCTCGTTTCTTTTTCTTCGTCGAAAAAGGCAATAAAAATTTTTATCACGTTTCTT
TTTCTTGAAAATTTTTTTTTTTGATTTTTTTCTCTTTCGATGACCTCCCATTGATATTTAAGTTAATAAACGGTCTTCAATTTCT
CAAGTTTCAGTTTCATTTTTCTTGTTCTATTACAACTTTTTTTACTTCTTGCTCATTAGAAAGAAAGCATAGCAATCTAATCTA
AGTTTTAATTACAAGGATCC**ATGCAAATATTATTGGCGAATCCTAGAGGGTTCTGTGCTGGCGTCGACAGGGCCATCAGT**
ATAGTGGAAAATGCTTTAGCAATTTATGGGGCCCCTATATACGTAAGACACGAAGTCGTTCATAATAGATATGTTGTAG
ATAGTTTGAGAGAAAGAGGAGCAATTTTTATCGAACAAATTTCGGAAGTGCCTGACGGGGCTATCTTAATTTTCTCAGC
CCATGGCGTATCCCAAGCCGTTAGAAATGAGGCAAAGTCGAGAGATTTGACGGTCTTCGATGCGACATGTCCTCTAGTG
ACAAAGGTTCATATGGAGGTGGCTCGTGCTTCCCGTAGAGGTGAAGAGAGTATTCTTATAGGACACGCAGGTCATCCTG
AAGTCGAAGGAACCATGGGCCAATATTCAAATCCAGAAGGTGGTATGTATTTGGTCGAATCTCCCGACGACGTCTGGAA
GCTAACTGTCAAAAATGAAGAAAAGTTGAGCTTTATGACCCAAACAACACTATCGGTAGATGATACCAGTGACGTGATT

GACGCGTTAAGAAAAAGGTTCCCGAAGATCGTTGGTCCCAGAAAGGATGACATTTGTTACGCCACTACTAACAGACAA
GAAGCGGTGCGTGCTTTAGCTGAGCAAGCAGAGGTTGTTTTGGTGGTTGGTTCTAAAAATAGTAGTAATTCCAATAGGT
TAGCTGAATTGGCTCAAAGGATGGGCAAGAGGGCGTTTCTAATTGATGATGCGAAAGATATACAAGAGGAGTGGGTC
AAAGAAGTTAAATGCGTGGGAGTTACTGCTGGCGCATCTGCCCCAGACATCCTAGTGCAGAACGTTGTTGCAAGGTTAC
AACAATTGGGTGGTGGTGAGGCCATCCCATTAGAAGGTAGAGAAGAAAACATAGTGTTTGAAGTGCCAAAAGAATTG
AGGGTGGATATACGTGAGGTCGATTGAATCCGCTCTAACCGAAAAGGAAGGAGTTAGACAACCTGAAGTCTAGGTCCCT
ATTTATTTTTTTATAGTTATGTTAGTATTAAGAACGTTATTTATATTTCAAATTTTTCTTTTTTTTCTGTACAGACGCGTGTACG
CATGTAACATTATACTGAAAACCTTGCTTGAGAAGGTTTTGGGACGCTCGAAGTTTTAGAATATATCTCTCCAATACAGCGT
TACCAATATGGTATGCCGAGTCTTGGGTTGCCAAACTAAGAGGCCATGGAATATATTTGAATGTTTGTGATTTGGCTTCATT
GTAACATGTAAGTGAACATCAAAAGAGTAGGCATTAAAAGATGGGACGTCAGCACTGTACTTGTTTTTGCGACTAGATTGT
AAATCATTCTTTATTTAATCTCTTTCTTTAACTACTGCTTAAAGTATAATTTGGTCCGTAGTTTAATAACTATACTAAGCGTAA
CAATGCATACTGACATTATAAGCCTGAACATTACGAGTTTAAGTTGTATGTAGGCGTTCTGTAAGAGGTTACTGCGTAAAT
TATCAACGAATGCATTGGTGTATTTGCGAAAGCTACTTCTTTTAACAAGTATTTACATAAGAATAATGGTGATCTGCTCAAC
TGATTTGGTGATAACTCTAACTTTTTTAGCAACAATTTAAAAGATAATTCGAACATATATAGGCGCGCC
